# Construction of Four Zn(II) Coordination Polymers Used as Catalysts for the Photodegradation of Organic Dyes in Water

**DOI:** 10.3390/polym8010003

**Published:** 2016-01-06

**Authors:** Lei-Lei Liu, Cai-Xia Yu, Wei Zhou, Qi-Gui Zhang, Shi-Min Liu, Yun-Feng Shi

**Affiliations:** College of Chemistry and Chemical Engineering, Anyang Normal University, Anyang 455000, China; yucaixiachem@163.com (C.-X.Y.); wisabc@163.com (W.Z.); yzh007118@sina.com (Q.-G.Z.); liushimin3495@163.com (S.-M.L.)

**Keywords:** coordination polymers, photocatalytic properties, crystal structures, solvothermal reactions

## Abstract

Hydrothermal reactions of Zn(OAc)_2_·2H_2_O with flexible bipyridyl benzene ligand and three dicarboxylic derivatives gave rise to four new coordination polymers, [Zn_7_(μ_4_-O)_2_(OAc)_10_(bpmb)]*_n_* (**1**), [Zn(5-OH-1,3-BDC)(bpmb)]*_n_* (**2**), [Zn(1,2-BDC)(bpmb)]*_n_* (**3**) and [Zn_2_(ADB)_2_(bpmb)]*_n_* (**4**) (bpmb = 1,4-bis(pyridine-3-ylmethoxy)benzene, 5-OH-1,3-H_2_BDC = 5-hydroxy-1,3-benzenedicarboxylic acid, 1,2-H_2_BDC = 1,2-benzenedicarboxylic acid, H_2_ADB = 2,2’-azodibenzoic acid). Their structures were characterized by single-crystal X-ray diffraction, elemental analyses, IR spectra, powder X-ray diffraction (PXRD) and thermogravimetric analyses (TGA). Compound **1** features a one-dimensional (1D) chain structure based on the rare heptanuclear [Zn_7_(μ_4_-O)(μ_3_-OAc)_2_(μ_2_-OAc)_8_] units. Compound **2** exhibits a novel 2D bilayer structure built from the two parallel 2D (4,4) layers. Compound **3** holds a 2D structure in which the 1,2-BDC ligands work as lockers interlocking 1D [Zn(bpmb)]*_n_* chain. Compound **4** comprises a 3D framework constructed by 2D wrinkled [Zn_2_(ADB)_4_]*_n_* networks and bpmb linkers with a six-connected pcu net. These results suggest that the motifs of the dicarboxylic ligands have significant effect on the final structures. These compounds exhibited relatively good photocatalytic activity towards the degradation of methylene blue (MB) in aqueous solution under a Xe lamp irradiation.

## 1. Introduction

In recent years, increasing attention on functional coordination polymers (CPs) has led to the fast development of this type of solid material, which are due to their intriguing aesthetic structures and topological features, as well as their potential applications in catalysis, adsorption, separation and so on [1,2,3,4,5,6,7,8,9,10,11,12]. Significant effort has been devoted to producing CPs with desired structures and properties using various approaches [13]. Practically, a variety of examples have demonstrated that the physical and chemical properties of the linkers play a decisive role in the structures and functions of novel CPs [14,15,16]. Many flexible bipyridyl ligands, such as 1,4-bis(pyridine-2-ylmethoxy)benzene, 1,3-bis(pyridine-3-ylmethoxy)benzene, and 1,2-bis(pyridine-4-ylmethoxy)benzene have been employed to construct varied functional CPs [17,18,19]. Such flexible ligands could adopt various conformations and make changeable CPs [20,21].

Photocatalysis is a green technology for the treatment of all kinds of contaminants that has many advantages over other treatment methods; for instance, the use of the environmentally friendly oxidant (O_2_ or H_2_O_2_), the ambient temperature reaction condition, and oxidation of the organic compounds, even at low concentrations [22,23]. Recently, considering the novelty of this field in CPs, much effort has been devoted to developing new photocatalytic materials based on CPs in the degradation of many kinds of organic contaminants with up to 90% efficiency [24,25,26,27,28]. Compared to the traditional semiconductor metal oxide, the advantages of CPs as photocatalyst lie in the fact that their combination of inorganic and organic moieties results in different metal–ligand charge transfer, which can give rise to tunable photocatalysts [29]. Lately, some metal CPs corresponding to the Cd(II), Cu(II) and Mn(II) ions, have been reported to be active in catalyzing the photodegradation of organic dyes [30,31,32,33,34]. However, the exploration of Zn(II)-based coordination complexes as effective photocatalysts is relatively rare [35,36,37].

We have been interested in the construction of CPs derived from metal ions and bridging *N*- or *O*- donor ligands [38,39,40]. Some of them could efficiently catalyze the photodegradation of organic dyes [41]. Aiming to search for more effective photocatalysts, four new CPs, [Zn_7_(μ_4_-O)_2_(OAc)_10_(bpmb)]*_n_* (**1**), [Zn(5-OH-1,3-BDC)(bpmb)]*_n_* (**2**), [Zn(1,2-BDC)(bpmb)]*_n_* (**3**) and [Zn_2_(ADB)_2_(bpmb)]*_n_* (**4**), were successfully synthesized by the starting materials flexible bipyridyl ligand 1,4-bis(pyridine-3-ylmethoxy)benzene (bpmb) and Zn(OAc)_2_·2H_2_O together with different rigid/flexible dicarboxylic auxiliary ligands 5-OH-1,3-H_2_BDC, 1,2-H_2_BDC and H_2_ADB under solvothermal conditions. These four CPs were found to be able to photocatalytically degrade methylene blue (MB) in water in a relatively efficient way.

## 2. Experimental Section

### 2.1. Chemicals and Characterization

The ligand bpmb was prepared according to the previously reported procedure with modification [38]. All other chemicals and reagents were obtained from commercial sources and used as received. Infrared (IR) spectra were recorded with a Varian 800 Fourier transform infrared (FT-IR) spectrometer (Varian, Inc., Palo Alto, CA, USA) as KBr disks (4000–400 cm^−1^). The elemental analysis for C, H, and N was performed on an EA1110 CHNS elemental analyzer (Carlo Erba, Inc., Milan, Italy). Powder X-ray diffraction (PXRD) was performed using a PANalytical X’Pert3 Powder instrument (PANalytical B.V., Almelo, The Netherlands) with Cu Kα radiation. Thermal gravimetric (TG) analysis was performed on a NETZSCH STA-449F3 instrument (Netzsch, Co., Selb, Germany) in flowing N_2_ with a heating rate of 10 °C·min^−1^, coupled with a Bruker TENSOR27 Fourier Transform Infrared Spectrometer (Bruker Optics, Inc., Ettlingen, Germany).

### 2.2. Synthesis

#### 2.2.1. Synthesis of Compound **1**

[Zn_7_(μ_4_-O)_2_(OAc)_10_(bpmb)]*_n_* (**1**). A 10 mL Pyrex glass tube was loaded with Zn(OAc)_2_·2H_2_O (9 mg, 0.04 mmol), bpmb (6 mg, 0.02 mmol) and 4 mL of MeCN. The tube was then sealed and heated in an oven to 150 °C for four days, and then cooled to ambient temperature at a rate of 5 °C·h^−1^. The colorless blocks of **1** were formed four day later, which were collected and dried in air. Yield: 8 mg (29%, based on bpmb). Anal. Calcd. for C_38_H_46_N_2_Zn_7_O_24_: C, 33.25; H, 3.38; N, 2.04. Found: C, 33.58; H, 3.55; N, 1.89. IR (KBr disc): 3378 (m), 2963 (w), 1612 (m), 1590 (s), 1506 (m), 1438 (m), 1403 (s), 1341 (m), 1231 (m), 1108 (w), 1052 (m), 1023 (m), 933 (w), 862 (w), 823 (w), 797 (w), 780 (w), 700 (m), 656 (m)·cm^−1^.

#### 2.2.2. Synthesis of Compound **2**

[Zn(5-OH-1,3-BDC)(bpmb)]*_n_* (**2**). A mixture of Zn(OAc)_2_·2H_2_O (9 mg, 0.04 mmol), bpmb (6 mg, 0.02 mmol), 5-OH-1,3-H_2_BDC (4 mg, 0.02 mmol), and 4 mL of H_2_O was sealed in a 10 mL Pyrex glass tube and heated at 170 °C for four days, then cooled to room temperature at a rate of 5 °C·h^−1^. The colorless blocks of **2** were collected and dried in air. Yield: 6 mg (56%, based on bpmb). Anal. Calcd. for C_26_H_20_N_2_ZnO_7_: C, 58.06; H, 3.75; N, 5.21. Found: C, 58.38; H, 3.55; N, 5.51. IR (KBr disc): 3300 (m), 3081 (w), 1627 (m), 1575 (s), 1508 (s), 1436 (m), 1400 (m), 1381 (m), 1349 (m), 1271 (m), 1237 (s), 1199 (m), 1107 (w), 1055 (m), 1000 (w), 831 (w), 794 (m), 782 (m), 728 (w), 700 (m), 656 (w)·cm^−1^.

#### 2.2.3. Synthesis of Compound **3**

[Zn(1,2-BDC)(bpmb)]*_n_* (**3**). Compound **3** (colorless rods) was prepared in the same way as **2**, except using 1,2-H_2_BDC (3 mg, 0.02 mmol) instead of 5-OH-1,3-H_2_BDC. Yield: 5 mg (48%, based on bpmb). Anal. Calcd. for C_26_H_20_N_2_ZnO_6_: C, 59.84; H, 3.86; N, 5.39. Found: C, 59.90; H, 3.60; N, 5.60. IR (KBr disc): 3465 (m), 3073 (w), 1610 (m), 1508 (m), 1562 (s), 1442 (m), 1390 (m), 1271 (w), 1234 (m), 1213 (m), 1192 (w), 1128 (w), 1049 (m), 1023 (m), 798 (m), 749 (m), 708 (m), 654 (w)·cm^−1^.

#### 2.2.4. Synthesis of Compound **4**

[Zn_2_(ADB)_2_(bpmb)]*_n_* (**4**). Compound 4 (orange blocks) was prepared in the same way as 2, except using H_2_ADB (6 mg, 0.02 mmol) instead of 5-OH-1,3-H_2_BDC and the reaction temperature was fixed at 150 °C. Yield: 7 mg (36%, based on bpmb). Anal. Calcd. for C_46_H_32_N_6_Zn_2_O_10_: C, 57.58; H, 3.36; N, 8.76. Found: C, 57.79; H, 3.80; N, 8.57. IR (KBr disc): 3358 (m), 2928 (w), 1637 (s), 1581 (m), 1507 (m), 1411 (m), 1326 (w), 1228 (w), 1216 (w), 1119 (w), 1022 (m), 864 (w), 830 (m), 770 (m), 717 (w), 664 (w)·cm^−1^.

### 2.3. X-Ray Data Collection and Structure Determination

Single crystals of **1**–**4** were obtained directly from the above preparations. All measurements were made on a Bruker Smart Apex-II CCD area detector by using graphite monochromated Mo Kα (λ = 0.071073 nm). These crystals were mounted on glass fibers at 296 K for **1**–**4**. Diffraction data were collected at *f* and ω modes with a detector distance of 35 mm to the crystals. Cell parameters were refined using the program Bruker *SAINT*. The collected data were reduced using the program Bruker *SAINT* A, and the absorption corrections (multi-scan) were applied. The reflection data were also corrected for Lorentz and polarization effects. The crystal structures of **1**–**4** were solved by direct method refined on *F*^2^ by full-matrix least-squares techniques with the SHELXTL-97 program [42]. A summary of the key crystallographic information for **1**–**4** is tabulated in Table 1.

**Table 1:** Summary of crystallographic data for **1**–**4**.

Compounds	1	2	3	4
Empirical formula	C_38_H_46_N_2_O_24_Zn_7_	C_26_H_20_N_2_O_7_Zn	C_26_H_20_N_2_O_6_Zn	C_23_H_16_N_3_O_5_Zn
Formula weight	1372.50	537.83	521.81	479.76
Crystal system	Triclinic	Triclinic	Monoclinic	Triclinic
Space group	*P*ī	*P*ī	*P*2_1_	*P*ī
*a*/Å	10.231(2)	9.6355(19)	9.4819(19)	10.259(2)
*b*/Å	11.627(2)	9.950(2)	10.746(2)	10.563(2)
*c*/Å	12.602(3)	13.936(3)	11.348(2)	10.846(2)
α/°	114.18(3)	78.20(3)	90.00	106.60(3)
β/°	110.45(3)	84.53(3)	92.56(3)	96.99(3)
γ/°	91.34(3)	61.85(3)	90.00	109.45(3)
*V*/Å^3^	1256.9(6)	1153.2(4)	1155.1(4)	1030.9(4)
*Z*	1	2	2	2
Temperature/K	296(2)	296(2)	296(2)	296(2)
*D*_c_/g·cm^−3^	1.813	1.549	1.500	1.545
μ/Mo Kα, mm^−1^	3.366	1.117	1.109	1.233
*F*(000)	690.0	552.0	536.0	490.0
Total reflections	9665	8065	8178	7268
Unique reflections(*R*_int_)	4406(0.0206)	4054(0.0430)	3961(0.0198)	3618(0.0647)
No. of observations	3784	3189	3758	2601
No. of parameters	327	325	317	289
*R*_1_^a^	0.0282	0.0416	0.0232	0.0485
*wR*_2_^b^	0.0710	0.1185	0.0525	0.1379
*GOF* ^c^	1.030	1.054	1.038	1.036

^a^ *R*_1_ = Σ||*F*_o_| − |*F*_c_|/Σ|*F*_o_|; ^b^ *wR*_2_ = {Σ*w*(*F*_o_^2^ − *F*_c_^2^)^2^/Σ*w*(*F*_o_^2^)^2^}^1/2^; ^c^ GOF = {Σ*w*(*F*_o_^2^ − *F*_c_^2^)^2^/(*n* − *p*)}^1/2^, where *n* = number of reflections and *p* = total numbers of parameters refined.

### 2.4. Photocatalytic Activity Measurements

The photocatalytic activities of as-prepared samples were evaluated by the degradation of MB under irradiation of a 350 W Xe lamp with the whole spectrum. The as-synthesized single crystals obtained from the above preparations, which were further grinded into microcrystals with a size of about 70 μm (Figure S1). In a typical process, 20 mg grinded samples as photocatalysts were added into 50 mL of MB aqueous solution (4 × 10^−5^ mol·L^−1^). The MB aqueous solution was stirred for 30 min in the dark before irradiation to reach adsorption equilibrium between the catalyst and solution and then it was exposed to the Xe lamp irradiation. About 4 mL suspension was continually taken from the reaction cell and collected by centrifugation at each 30 min interval during irradiation. The resulting solution was analyzed on a Varian 50 UV–Vis spectrophotometer (Varian, Inc., Palo Alto, CA, USA).

## 3. Results and Discussion

### 3.1. Synthetic and Spectral Aspects

**Figure 1:**
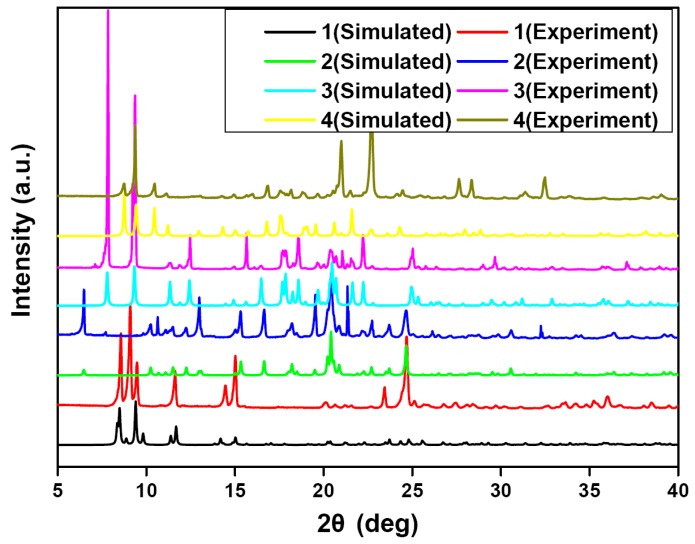
Experimental and simulated powder X-ray diffraction (PXRD) patterns for **1**–**4**.

Treatment of bpmb with Zn(OAc)_2_·2H_2_O in MeCN followed by a hydrothermal condition at 150 °C for four days produced crystals of **1** (29% yield). Furthermore, similar reactions of Zn(OAc)_2_·2H_2_O with bpmb and dicarboxylic derivatives at 170 °C (**2**–**3**) and 150 °C (**4**) in water generated crystals of **2** (56% yield), **3** (48% yield) and **4** (36% yield), respectively. When the reaction temperatures were decreased to 120 °C, only precipitates were isolated and their PXRD patterns were inconsistent with those of **1**–**4**. Compounds **1**–**4** were stable towards oxygen and moisture, and almost insoluble in common organic solvents. Their elemental analyses were consistent with the chemical formulas of **1**–**4**. In order to check the phase purity of **1**–**4**, the powder X-ray diffraction (PXRD) patterns were measured at room temperature (Figure 1). The identities of **1**–**4** were finally confirmed by single-crystal diffraction analysis.

### 3.2. Crystal Structure of **1**

**Figure 2:**
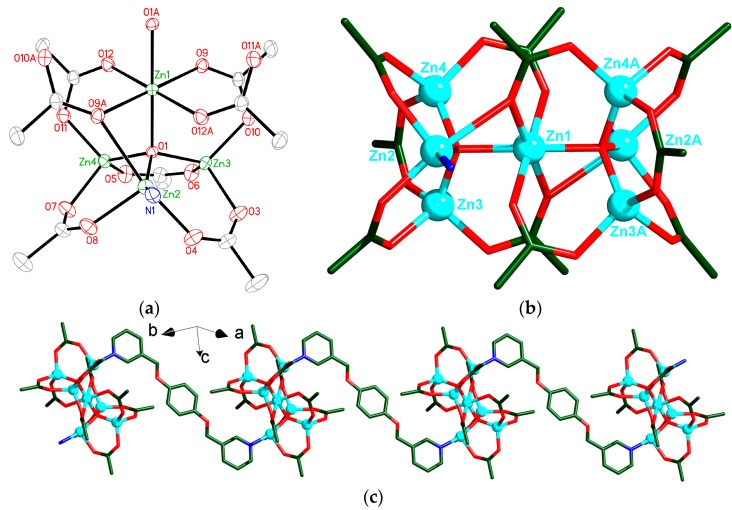
(**a**) View of the coordination environments of Zn centers in **1** with labeling schemes. Symmetry codes: (A) 1 − *x*, 1 − *y*, 1 − *z*. (**b**) View of one heptanuclear [Zn_7_(μ_4_-O)(μ_3_-OAc)_2_(μ_2_-OAc)_8_] unit of **1**. Symmetry codes: (A) 1 − *x*, 1 − *y*, 1 − *z*. (**c**) View of the 1D chain in **1** extending along the *a*-axis. Atom color codes: Zn, cyan; O, red; N, blue; C, dark green. All H atoms are omitted for clarity.

Compound **1** crystallizes in the triclinic space group *P*ī, and its asymmetric unit contains three and a half crystallographically independent Zn atoms, one μ_4_-O group, five OAc**^−^** ligands and a half bpmb ligand. As shown in Figure 2a, Zn1 atom adopts an octahedral coordination geometry and is six-coordinated by two O atoms of two μ_4_-O groups, four O atoms of four bridging carboxylate groups from four OAc**^−^** anions. While each Zn2 atom adopts a trigonal bipyramidal coordination geometry, coordinated by one O atom of μ_4_-O group, four O atoms of four bridging carboxylate groups from four OAc**^−^** anions and one N atom from one bpmb ligand (Figure 2a). Zn3 and Zn4 atoms are tetrahedrally coordinated by four O atoms from one μ_4_-O group and three bridging OAc**^−^** anions (Figure 2a). Zn1, Zn2, Zn3, Zn4 and its symmetry-related Zn2A, Zn3A and Zn4A are bridged by two μ_4_-O atoms and ten bridging OAc**^−^** groups to generate a heptanuclear [Zn_7_(μ_4_-O)(μ_3_-OAc)_2_(μ_2_-OAc)_8_] unit (Figure 2b). The Zn∙∙∙Zn separations in heptanuclear unit are 3.035–3.217 Å. The heptanuclear [Zn_7_(μ_4_-O)(μ_3_-OAc)_2_(μ_2_-OAc)_8_] units are further linked by bpmb ligands to form a 1D chain extending along the *a*-axis (Figure 2c).

### 3.3. Crystal Structure of **2**

**Figure 3:**
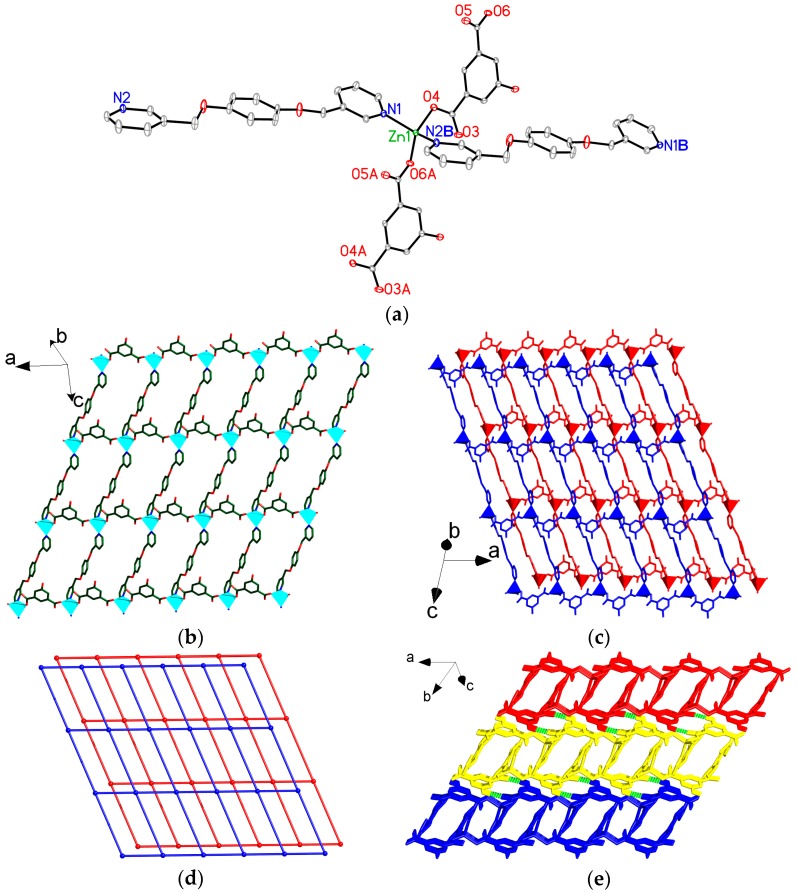
(**a**) View of the coordination environments of Zn center in **2** with labeling schemes. Symmetry codes: (A) − 1 + *x*, 1 + *y*, − 1 + *z*; (B) 1 + *x*, *y*, *z*; and (C) − 1 + *x*, 1 − *y*, − 1/2 + *z*; (**b**) View of the 2D (4,4) layer in **2**; (**c**) View of the 2D bilayer structure in **2** looking down *b*-axis; (**d**) View of the 2D bilayer model in **2**. Each single net represents a topology with a Schläfli symbol of 4^4^6^2^; (**e**) View of a 3D supramolecular framework in **2**. Green dashed lines represent the hydrogen-bonded interactions. Atom color codes: Zn, cyan polyhedrons; O, red; N, blue; C, dark green and pink. All hydrogen atoms except those related to H-bonding interactions are omitted for clarity.

Compound **2** crystallizes in the monoclinic space group *P*2*/c*, its asymmetric unit contains one [Zn(5-OH-1,3-BDC)(bpmb)] unit. Each Zn atom is coordinated by two N atoms from two different bpmb ligands and two O atoms of bridging carboxylate groups from two 5-OH-1,3-BDC ligands to complete the tetrahedral geometry (Figure 3a). Each Zn^II^ atom is interlinked by bis-monodentate 5-HO-1,3-BDC ligands to form a 1D [Zn(5-HO-1,3-BDC)]*_n_* chain extending along the *a*-axis (Figure 3b). Each chain is connected to adjacent chains *via* bpmb ligands to produce a 2D (4,4) layer (extending along the *ac* plane), with parallelogram-shaped meshes (9.635 Å × 16.363 Å, between Zn atoms at the corners) (Figure 3b). Interestingly, such 2D layer parallels to the equivalent one with a interleaving, resulting in a rare bilayer structure extending along the *ac* plane (Figure 3c,d). From the topological view [43], if the Zn centres are considered as nodes and the 5-HO-1,3-BDC and bpmb ligands are considered as linkers, the bilayer structure of **2** can be specified by a Schläfli symbol of 4^4^6^2^ (Figure 3d). Further investigation of the crystal packing of compound **2** suggests that each bilayer structure is interconnected with adjacent ones through intermolecular H-bonding interactions among the uncoordinated O atoms of the carboxylate groups from 5-HO-1,3-BDC ligands and the H atoms of the hydroxyl groups [O7–H7···O3, (− *x*, − *y* + 1, − *z*), 1.87(3) Å], which leads to the formation of a 3D supramolecular framework (Figure 3e).

### 3.4. Crystal Structure of **3**

**Figure 4:**
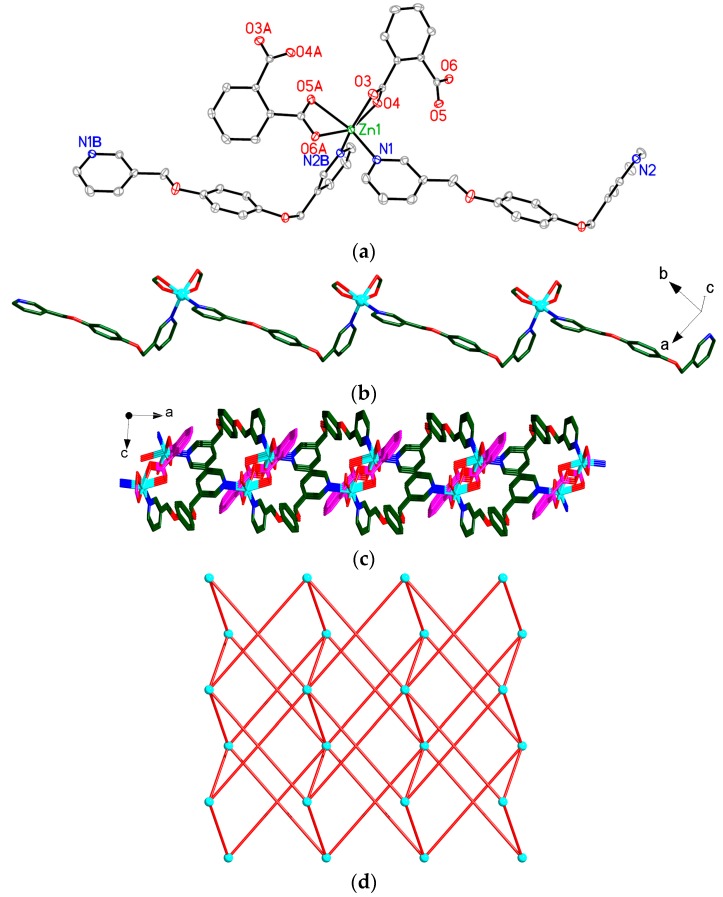
(**a**) View of the coordination environments of Zn center in **3** with labeling schemes. Symmetry codes: (A) 1 − *x*, 1/2 + *y*, 1 − *z*; (B) 1 + *x*, 1 + *y*, *z*; (**b**) View of the 1D [Zn(bpmb)]*_n_* chain in **3**; (**c**) View of the 2D structure in **3** looking down *b*-axis; (**d**) Schematic view of the six-connected 4^12^6^3^ net of **3**. Atom color codes: Zn, cyan; O, red; N, blue; C, dark green and pink. All H atoms are omitted for clarity.

Compound **3** crystallizes in the monoclinic space group *P*2_1_, and its asymmetric unit contains an independent [Zn(1,2-BDC)(bpmb)] molecule. Zn1 atom in **3** is six-coordinated by two N atoms (N1 and N2B) from two bpmb ligands, four O atoms (O3, O4, O5A and O6A) of two chelating carboxylate groups from two 1,2-BDC ligands (Figure 4a). As shown in Figure 4b, the Zn(II) ions are bridged by bpmb ligands generating a 1D [Zn(bpmb)]*_n_* chain. Interestingly, the 1,2-BDC ligands bond to Zn atoms in the up and down fashion in the 1D [Zn(bpmb)]*_n_* chain, producing a 2D structure extending along the *ac* plane (Figure 4c). Topologically, the overall structure of **3** can be described as a six-connected 4^12^6^3^ topology (Figure 4d).

### 3.5. Crystal Structure of **4**

**Figure 5:**
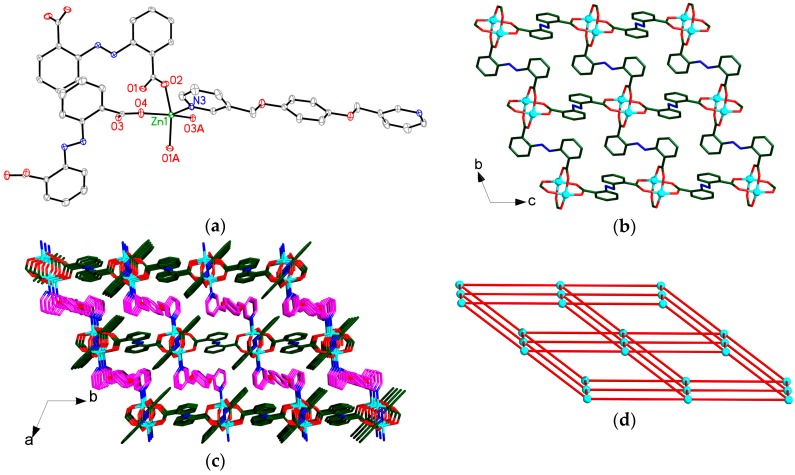
(**a**) View of the coordination environment of Zn center in **4** with labeling schemes. Symmetry codes: (A) 1 − *x*, 1 − *y*, 1 − *z*; (**b**) View of a wrinkled 2D network in **4** extending along the *bc* plane; (**c**) View of a 3D framework of **4** looking down the *c*-axis; (**d**) Schematic view of the six-connected *pcu* net of **4**. Atom color codes: Zn, cyan; O, red; N, blue; C, dark green and pink. All H atoms are omitted for clarity.

Compound **4** crystallizes in the triclinic space group *P*ī, and its asymmetric unit contains half of [Zn_2_(ADB)_2_(bpmb)] unit. The Zn1 atom adopts a pyramidal coordination geometry and is five-coordinated by four O atoms of four bridging carboxylate groups from four ADB ligands and one N atom of one bpmb ligand (Figure 5a). The Zn1 atom and its symmetry-related Zn1A atom are bridged by four carboxylate groups to generate a paddle-wheel [Zn_2_(μ_2_-CO_2_)_4_] unit (Figure 5b). The Zn∙∙∙Zn separation in this dinuclear unit is 2.9301 Å. Each paddle-wheel [Zn_2_(μ_2_-CO_2_)_4_] unit serves as a four-fold node, which links four equivalent ones *via* sharing of four ADB ligands to form a 2D wrinkled network extending along the *bc* plane (Figure 5b). Furthermore, the bpmb ligands are employed as linkers (pink) to bridge the 2D networks producing a 3D framework (Figure 5c). Topologically, the overall structure of **4** can be described as a *pcu* net with the six-connected 4^12^6^3^ topology (Figure 5d).

The structures of **1**–**4** are different in the following aspects. Firstly, Zn atoms in **1** are surrounded by four, five and six O atoms, exhibiting the trigonal pyramidal, trigonal bipyramidal and octahedral coordination geometries, respectively, while in **2**–**4**, Zn atoms only show one type of coordination geometry, namely, trigonal pyramid for **2**, distorted octahedron for **3** and trigonal bipyramid for **4**. Secondly, the bpmb ligands in **1**–**2** and **4** are employed as the linkers connecting the 0D heptanuclear [Zn_7_(μ_4_-O)(μ_3_-OAc)_2_(μ_2_-OAc)_8_] units (**1**), 1D [Zn(5-HO-1,3-BDC)]*_n_* chains (**2**) and 2D [Zn_2_(ADB)_4_]*_n_* networks (**4**) to produce the higher dimensional structures. In **3**, the bpmb ligands are connected by Zn atoms, resulting in a 1D [Zn(bpmb)]*_n_* chain. Thirdly, the carboxylate groups of the ancillary ligands display μ_2_-η^1^:η^1^ and μ_3_-η^1^:η^2^ coordination modes in **1**, μ_1_-η^1^:η^0^ in **2**, μ_1_-η^1^:η^1^ (**3**) and μ_2_-η^1^:η^1^ in **4**. In **1**, the OAc**^−^** anions work as the terminal ligands joining the seven Zn atoms to afford the heptanuclear [Zn_7_(μ_4_-O)(μ_3_-OAc)_2_(μ_2_-OAc)_8_] unit. In **4**, the ADB ligands serve as two-connectted nodes to link Zn_2_ subunits forming 2D network, which may be due to the species of ancillary ligands. Fourthly, in **2**, the OH groups at the five-position of 1,3-BDC ligands act as hydrogen-bonding donors expand the 2D bilayers into the 3D hydrogen-bonded framework. While in **3**, as the H atom of 1,2-BDC ligand acts neither as a hydrogen-bonding donor nor as a hydrogen-bonding acceptor, it could not induce any hydrogen-bonding interactions. Therefore, the substituted groups in BDC ligands play important roles in determining the structures of **2**–**3**. From the above-mentioned comparison, it is noted that the species of ancillary ligands in this study greatly affected the formation of different coordination geometries of Zn(II) atoms, the conformations of the bpmb ligands and the whole structures of these compounds.

### 3.6. Thermal Property

**Figure 6:**
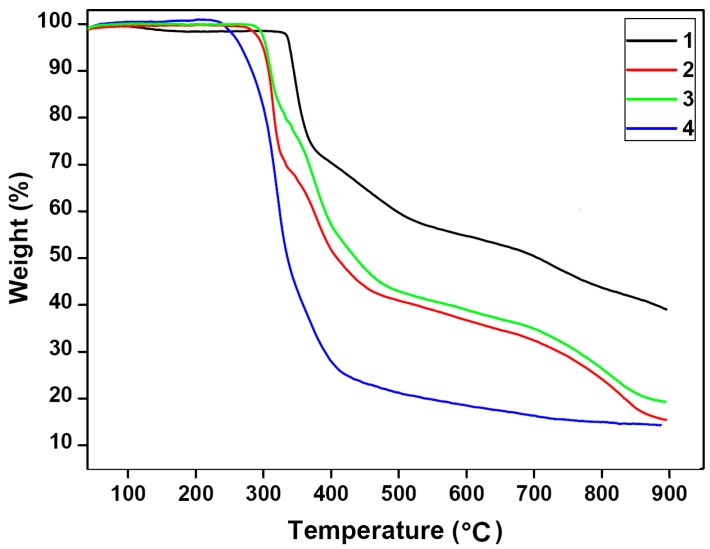
The thermogravimetric analyses (TGA) curves for **1**–**4**.

Thermogravimetric (TGA) experiments were carried out to study the thermal stability of **1**–**4**. As shown in Figure 6, the TGA curves of **1**–**4** show similar profiles. They are stable up to 326 °C for **1**, 285 °C for **2**–**3** and 235 °C for **4**, followed by the collapse upon further calcinations. The final residue of 40.27%, 16.72%, 18.90% and 14.55% for **1**–**4**, respectively, is in agreement with the percentage of ZnO (calculated 41.52%, 15.14%, 15.60% and 16.97%), indicating that this is the final product.

### 3.7. Photocatalytic Activity

**Figure 7:**
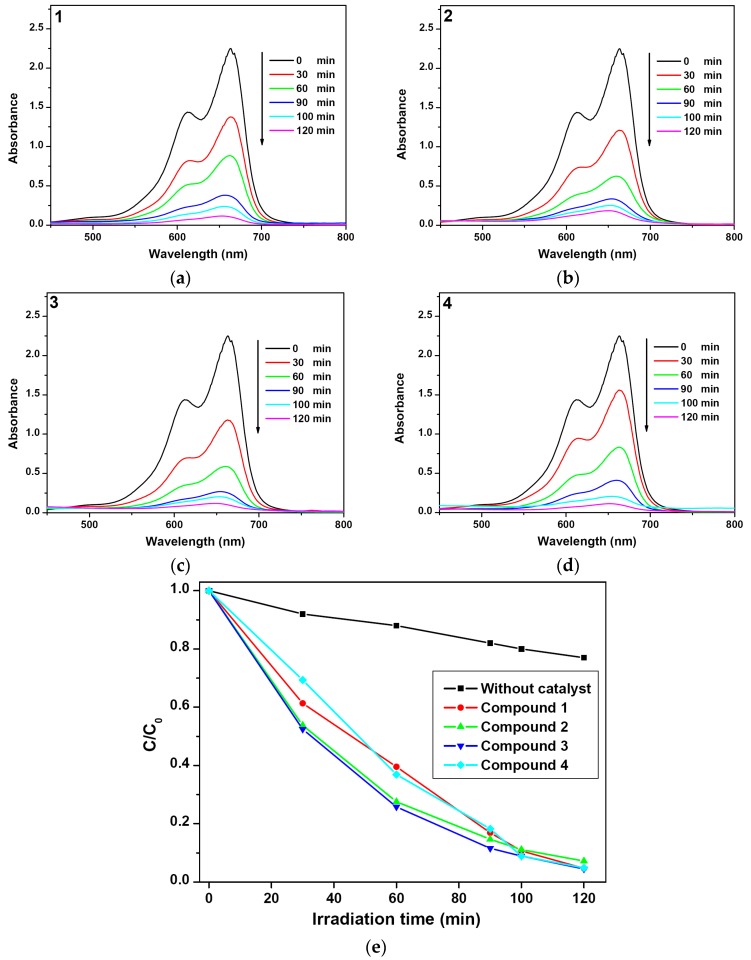
(**a**–**d**) Absorption spectra of the MB solution (4 × 10^−5^ mol·L^−1^, 50 mL) during the decomposition reaction under the Xe lamp irradiation with the presence of compounds **1**–**4** (20 mg); (**e**) Concentration changes of MB at different time intervals under Xe lamp irradiation with **1**–**4** as catalysts and without catalyst.

As is well-known, organic dyes such as MO, MB and RhB that were extensively used in the textile industry have been found to be toxic, stable and difficult to biodegrade. Thus, it is urgent to decompose such dye molecules into some simple molecules to reduce the environment pollution. Hence, in this work, the photocatalytic activities of the compounds **1**–**4** were evaluated by the degradation of MB under irradiation at room temperature as the test pollutant. To evaluate the band gaps, the UV–vis absorption spectrum of **1**–**4** is measured at room temperature (Figure S2). The results give *E*_g_ (band-gap energy) values of 3.46, 3.56, 3.62 and 3.64 eV for **1**–**4**, respectively (Figure S3). As illustrated in Figure 7a–d, the absorption peaks of MB in water decreased patently following the reaction time in the presence of **1**–**4**. The concentrations of organic dye were estimated by the absorbance at 665 nm (MB, absorption coefficient: 5.53 × 10^4^ L·mol^−1^·cm^−1^). The degradation efficiencies are defined as *C*/*C*_0_, where *C* and *C*_0_ represent the remnant and initial concentration of MB, respectively (Figure 7e). By contrast, the simple photolysis experiment was also performed under the same conditions without any catalyst. A comparison of the photocatalytic activities of **1**–**4** was presented in Figure 7e. The calculation results demonstrate that the photocatalytic activities increase from 23.0% (controlled experiment without any catalyst) to 95.3% for **1**, 92.8% for **2**, 95.5% for **3** and 95.2% for **4** after 120 min of irradiation, which are better than the commercial Degussa P25 TiO_2_ reference catalyst (84%), the pure ZnO (74%) and the ZnO@ZIF-8 materials [44]. Even compound **1** holds the narrowest *E*_g_ among these compounds, however, it exhibits almost same photocatalytic activity compared with compounds **3** and **4**, which may be due to the heptanuclear units in compound **1** that hinder the migration of excited electrons/holes and slow down the photocatalytic degradation process [34]. It is clear that compound **2** possesses lower activity than other compounds, which may be ascribed to the bilayer structures in **2**, leading to the MB molecules inaccessibility of the zinc centers. The catalyst was filtered and obtained a colorless solution, which was extracted by acetic ether, and the organic phase was analyzed by gas chromatography-mass spectrometer. No corresponding species of MB was observed, and thus we assumed that the dyes might be degradation of CO_2_ and H_2_O [22,45].

And the photocatalytic efficiencies of these compounds are comparable to those of other Zn-based CP materials. Such as, using the known CPs {[Zn_2_(Tipa)(4,4′-bpdc)_1.5_(H_2_O)(NO_3_)]·2(DMF)·H_2_O}*_n_* (Tipa = tris(4-(1H-imidazol-1-yl)-phenyl)amine, 4,4′-bpdc = 4,4′-biphenyldicarboxylate) [36] and {[Zn_2_(H_2_O)(1,4-ndc)_2_(tpcb)]}*_n_* (1,4-H_2_ndc = 1,4-naphthalenedicarboxylic acid, tpcb = tetrakis(4-pyridyl)cyclobutane) [35] as catalysts, which could degrade most of the MB with a relatively long times (240 and 600 min). Compared with other Cd-based CP materials, {[Cd(tpcb)_0.75_(OH)(H_2_O)_2_](NO_3_)}*_n_* [45], {[Cd(btbb)_0.5_(btec)_0.5_(H_2_O)]·2H_2_O}*_n_* (btbb = 1,4-bis(2-(4-thiazolyl)benzimidazole-1-ylmethyl)benzene, H_4_btec = 1,2,4,5-benzenetetracarboxylate) [28] and {[Cd_3_(bcb)_2_(H_2_O)_5_]·H_2_O}*_n_* (H_3_bcb = 3,4-bi(4-carboxyphenyl)-benzoic acid) [26] as catalysts, *ca.* 82.0%, 92.7% and 88.7% of MB was degraded in 120, 140 and 180 min, respectively. Combined with the UV–Vis adsorption spectra of **1**–**4** in the solid state (Figure S2), we inferred that the photocatalytic activities of these compounds could be attributed to the ZnO units. The valence and the conduction bands of ZnO are mainly due to O(2p) and Zn(4s) orbitals, respectively, this electronic transition can basically be described as an O^2−^Zn^2^^+^ → O^−^Zn^+^ LMCT. The organic linker acts as a photon antenna that could efficiently transfer the energy to the ZnO units [46]. In addition, the PXRD patterns of each powder for **1**–**4** were basically identical to those of the parent compounds, indicating that these compounds are stable during photocatalysis.

## 4. Conclusions

In summary, we demonstrated that treatment of Zn(OAc)_2_·2H_2_O with bpmb and dicarboxylic ligands with different motifs formed four different CPs **1**–**4**. These compounds exhibit various structural features. The photocatalytic activities of **1**–**4** were evaluated by the decomposition of organic dyes in aqueous solutions under the Xe lamp irradiation. These compounds showed good catalytic performance for the degradation of MB, which were excellent candidates for decomposing other organic dyes.

## Supplementary Materials

The following are available online at www.mdpi.com/2073-4360/8/1/3/s1. Figure S1 performs the SEM image of the grinded samples for **1**; Figure S2 presents the UV–vis adsorption spectra of **1**–**4** in the solid state at ambient temperature; Figure S3 shows the (Ahν)^2^–hν curves of **1**–**4**. Figure S4 displays the UV–vis adsorption spectra of the initial MB solution and the MB solution after stirring with the catalyst about 30 min.
