# Supplementary material for: Construction of Four Zn(II) Coordination Polymers Used as Catalysts for the Photodegradation of Organic Dyes in Water
Source: Polymers (Basel). 2016 Jan 6;8(1):3. doi: 10.3390/polym8010003 (PMC6432541; doi:10.3390/polym8010003)
Supplement: Supplementary file 1 [file polymers-08-00003-s001.pdf]

Supplementary Materials: Construction of Four Zn(II) Coordination Polymers Used as Catalysts for the Photodegradation of Organic Dyes in Water

Lei-Lei Liu, Cai-Xia Yu, Wei Zhou, Qi-Gui Zhang, Shi-Min Liu and Yun-Feng Shi

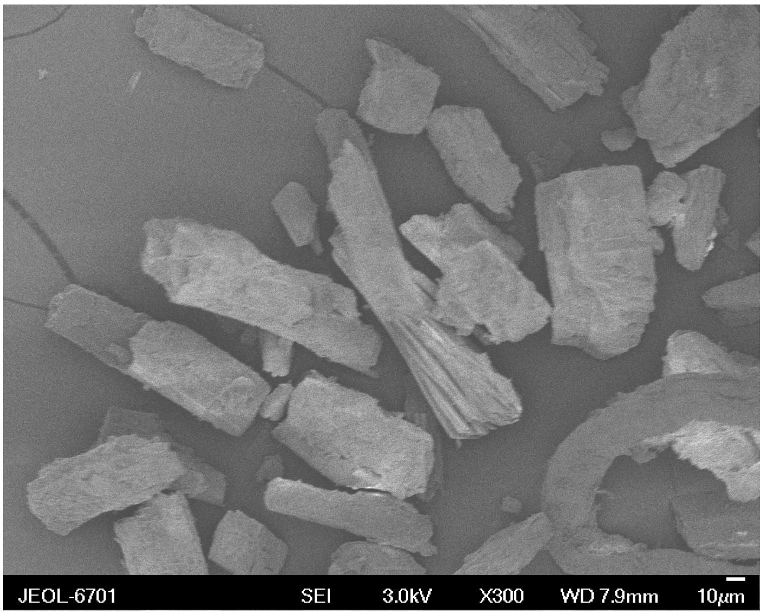

Figure S1. SEM image of the grinded samples for **1**.

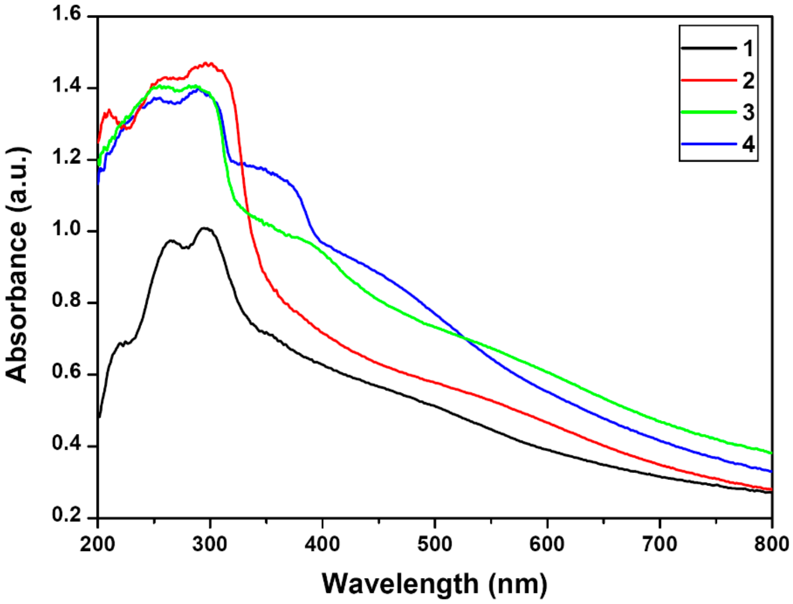

Figure S2. UV–vis adsorption spectra of **1**–**4** in the solid state at ambient temperature.

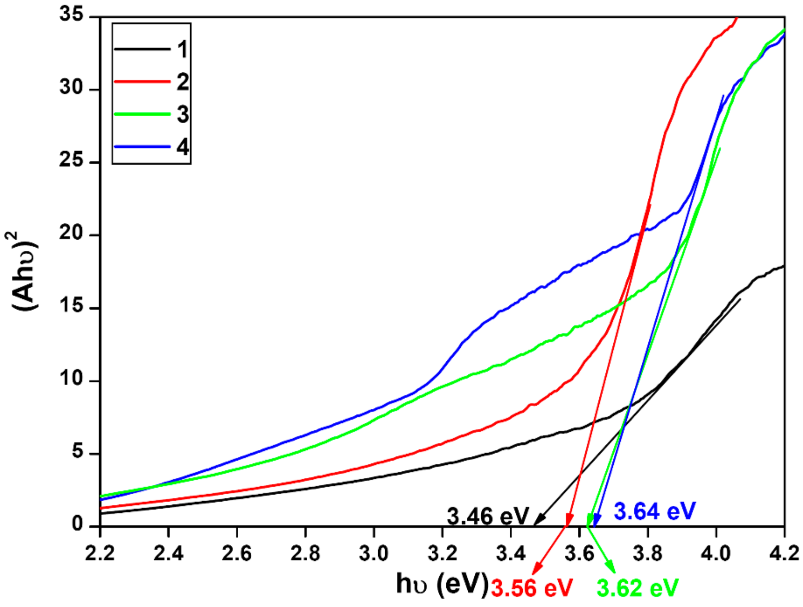

Figure S3. $(Ah\nu)^2$–$h\nu$ curves of **1**–**4**.

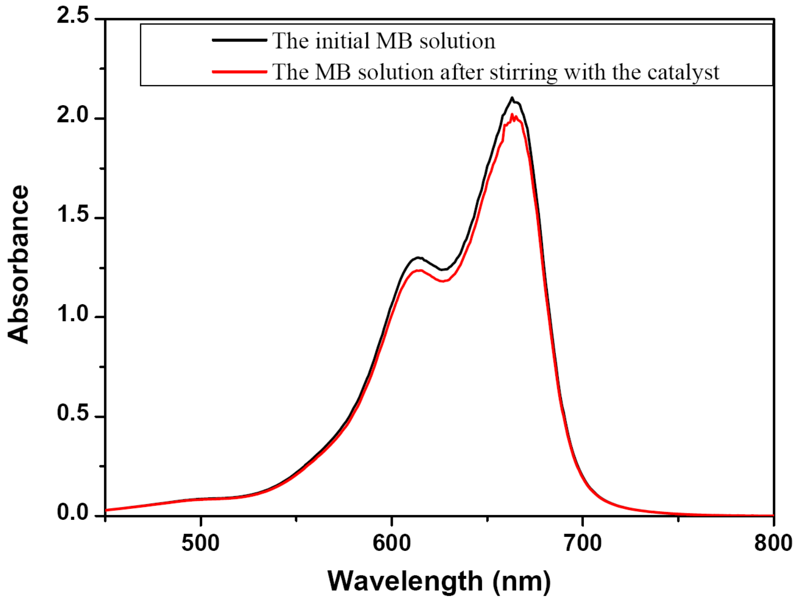

Figure S4. The UV–vis adsorption spectra of the initial MB solution and the MB solution after stirring with the catalyst about 30 min.

© 2016 by the authors; licensee MDPI, Basel, Switzerland. This article is an open access article distributed under the terms and conditions of the Creative Commons by Attribution (CC-BY) license (http://creativecommons.org/licenses/by/4.0/).
